# Validation of a tool to assess patient satisfaction, waiting times, healthcare utilization, and cost

**DOI:** 10.1017/S1463423619000094

**Published:** 2019-06-11

**Authors:** Breda H. Eubank, Mark R. Lafave, Nicholas G. Mohtadi, David M. Sheps, J. Preston Wiley

**Affiliations:** 1 Department of Health and Physical Education, Faculty of Health, Community, and Education, Mount Royal University, Calgary, AB, Canada; 2 Director Sport Medicine Centre, Faculty of Kinesiology, University of Calgary, Calgary, AB, Canada; 3 Division of Orthopaedics, Department of Surgery, University of Alberta, Edmonton, AB, Canada; 4 Sport Medicine Centre, Faculty of Kinesiology, University of Calgary, Calgary, AB, Canada

**Keywords:** patient satisfaction, psychometrics, quality of healthcare, rotator cuff disease, survey, waiting times

## Abstract

**Aim:**

Patients’ experience of the quality of care received throughout their continuum of care can be used to direct quality improvement efforts in areas where they are most needed. This study aims to establish validity and reliability of the Healthcare Access and Patient Satisfaction Questionnaire (HAPSQ) – a tool that collects patients’ experience that quantifies aspect of care used to make judgments about quality from the perspective of the Alberta Quality Matrix for Health (AQMH).

**Background:**

The AQMH is a framework that can be used to assess and compare the quality of care in different healthcare settings. The AQMH provides a common language, understanding, and approach to assessing quality. The HAPSQ is one tool that is able to assess quality of care according to five of six AQMH’s dimensions.

**Methods:**

This was a prospective methodologic study. Between March and October 2015, a convenience sample of patients presenting with chronic full-thickness rotator cuff tears was recruited prospectively from the University of Calgary Sport Medicine Centre in Calgary, Alberta, Canada. Reliability of the HAPSQ was assessed using test–retest reliability [interclass correlation coefficient (ICC)>0.70]. Validity was assessed through content validity (patient interviews, floor and ceiling effects), criterion validity (percent agreement >70%), and construct validity (hypothesis testing).

**Findings:**

Reliability testing was completed on 70 patients; validity testing occurred on 96 patients. The mean duration of symptoms was three years (SD: 5.0, range: 0.1–29). Only out-of-pocket utilization possessed an ICC<0.70. Patients reported that items were relevant and appropriate to measuring quality of care. No floor or ceiling effects were present. Criterion validity was reached for all items assessed. *A priori* hypotheses were confirmed. The HAPSQ represents an inexpensive, reliable, and valid approach toward collecting clinical information across a patient’s continuum of care.

## Background

Rotator cuff disorders (RCDs) ranks among the most prevalent of musculoskeletal disorders, yet treatment and management of these conditions are complex and a multitude of different treatment options exist (Yamaguchi, 2011). RCDs include a broad spectrum of acute and chronic pathological conditions, including tendinopathy, calcific tendinopathy, and rotator cuff tears. Of the three RCD pathologies, rotator cuff tears represent a significant proportion of RCDs, and are likely the most expensive in terms of public healthcare expenditure because many patients receive surgery as a means of treatment. In particular, patients with chronic rotator cuff tears suffer lengthy waiting times; inefficient use of healthcare resources, and disjointed care (Chehade *et al*., 2011; Frank *et al*., 2011; Lau *et al*., 2012; Mohtadi *et al*., 2012; Marshall *et al*., 2015). Measuring and analyzing data on quality of care would help to identify gaps in the current clinical pathway and suggest ways of improving care for patients presenting to the healthcare system with chronic rotator cuff tears.

Assessing healthcare quality is the first step to improving care and service delivery. Healthcare is a complex system that is often inefficient, error-prone, and costly (Buchert and Butler, 2016). Pressured to improve healthcare quality and economic efficiencies, physicians are often criticized for being less connected to patient needs, values, and preferences (Schippits and Schippits, 2013). Additionally, health decisions are becoming more complicated and patient care seems inconsistent with the availability of numerous clinical options (Schippits and Schippits, 2013). Consequently, specific strategies are needed for quality improvements through healthcare reform, which can result in patient-centered care, considerable savings of resources, and expansion of services for the community (Peacock *et al*., 2001). Measuring quality in healthcare is vital in evaluating patient outcomes and system performance before quality improvements can be achieved. Quality assessment can reveal the magnitude and nature of problems facing healthcare systems (Leatherman and Sutherland, 2010), and offers one method for evaluating the impact of changes to the organization and financing of healthcare services (McGlynn, 1997).

Measuring quality, however, is no simple task (McGlynn, 1997). Often, there is little systematic information about the extent to which standard processes involved in healthcare – a key element of quality – are delivered (McGlynn *et al*., 2003). Furthermore, there exists a gap between what works and what is actually done (McGlynn *et al*., 2003). Given the complexity and diversity of the healthcare system, there is no simple solution. A key component of any solution to measuring and reporting on quality is the availability of reliable and valid information on performance at all levels (McGlynn *et al*., 2003). Before quality can be measured, however, it must first be defined (Donabedian, 1988).

The Alberta Quality Matrix for Health (AQMH) is one approach to measuring, defining, and standardizing what quality healthcare means. It is an evidence-based approach that has been applied to other studies to assess and improve the quality of musculoskeletal care (Gooch *et al*., 2009; Frank *et al*., 2011; Schull *et al*., 2011). According to the AQMH, quality of care can be defined using six dimensions: accessibility, acceptability, efficiency, effectiveness, appropriateness, and safety (Health Quality Council of Alberta, 2003). Accessible health services are defined as those ‘obtained in the most suitable setting in a reasonable time and distance’ (Health Quality Council of Alberta, 2003). Acceptable health services are defined as being ‘respectful and responsive to user needs, preferences, and expectations’ (Health Quality Council of Alberta, 2003). Efficient health services are defined as ‘resources that are optimally used in achieving desired outcomes’ (Health Quality Council of Alberta, 2003). Effective health services are defined as being ‘based on scientific knowledge to achieve desired outcomes’ and refer to the efficacy of an intervention in providing the best outcome for the patient (Health Quality Council of Alberta, 2003). Appropriate health services are defined as being ‘relevant to user needs and are based on accepted or evidence-based practice’ (Health Quality Council of Alberta, 2003). In addition, safe health services are defined as being able to ‘mitigate risks to avoid unintended or harmful results’ (Health Quality Council of Alberta, 2003).

The AQMH is only a theoretical framework that defines six quality dimensions. Therefore, it only offers a common language, understanding, and approach to assessing quality in healthcare (Health Quality Council of Alberta, 2003). It is not a tool for gathering quantitative aspects of patients’ experience that can be used to evaluate care such as waiting times, patient satisfaction, health resource utilization, and care processes. Our group previously developed a tool for clinicians and healthcare teams to assess quality of care according to five of six AQMH’s dimensions – the Healthcare Access and Patient Satisfaction Questionnaire (HAPSQ) (Lau *et al*., 2012; Mohtadi *et al*., 2012). The HAPSQ does not assess effectiveness. To our knowledge, a search of the literature did not find any patient-report tools that were comparable and able to assess quality of care consistent with AQMH’s framework. Although the HAPSQ demonstrated good reliability and validity for patients with acute knee injuries (Lau *et al*., 2012; Mohtadi *et al*., 2012), reliability and validity are context and patient population-specific. Therefore, the psychometric properties of the HAPSQ were tested within the context of patients presenting to healthcare settings with chronic rotator cuff tears.

## Methods

### Early development

The HAPSQ was originally developed to measure the quality of care for patients presenting with acute knee injuries consistent with the AQMH (Lau, 2009). The early development of the HAPSQ occurred between 2006 and 2008 using patients recruited from the University of Calgary Sport Medicine Centre in Calgary, Alberta, Canada (Lau, 2009). The primary investigator (B.E.) initially generated a list of 39 fixed items. The initial item list was then circulated to a working group consisting of content experts and members of stakeholder groups. The working group examined the list for content validity and comprehensiveness, and modifications were made in response to the comments received. The revised list was then tested using expert focus groups and patient interviews. The HAPSQ experienced 19 iterations in which items were modified to improve clarity. HAPSQ (version 19) underwent reliability and validity testing (Lau, 2009). Items that failed to meet test–retest reliability or possessed little variance were discarded resulting in 29 fixed items. The HAPSQ (version 20) has since been used to evaluate the quality of care in patients presenting with acute knee injuries (Lau *et al*., 2012; Mohtadi *et al*., 2012). The HAPSQ (version 20) is a web-based questionnaire. The web-based interface provided several advantages over traditional survey methods in terms of cost, speed, appearance, flexibility, functionality, and usability. The HAPSQ (version 20) was adapted to patients presenting with chronic rotator cuff tears by modifying questions with the word ‘knee’ to ‘shoulder.’ All other aspects of item organization and wording remained unchanged.

### The HAPSQ

The HAPSQ is a self-administered, multipurpose web-based questionnaire that collects information related to healthcare utilization, access, and patient satisfaction. To ensure readability, the HAPSQ was designed at a 10th-grade reading level and the interface font was legible. A high-contrast design was created by using black text on a white background to ensure optimal legibility and esthetics (Hall and Hanna, 2004). Pop-up instructions and error messages eliminated nonresponse errors. Progressive indicators were also placed on the left-hand side of the screen to show respondents how far through the questionnaire they were. Individual items are grouped into sections of the questionnaire rather than scales: use of physician services (eg, GP/family physician, orthopedic surgeon) (four items); use of diagnostic investigations (three items); surgery (two items); use of complementary allied medical treatments (eg, physical therapy, massage therapy) (three items); out-of-pocket expenses (three items); lost wages (four items); patient satisfaction rating of care (two items); patient expectations around acceptable waiting times (one item); and demographic information (seven items). The HAPSQ has 29 fixed items, however, the total item count within several sections can vary depending on the quantity of services rendered or items purchased. For example, if the patient received care from two family physicians and one surgeon, then the number of items in that section would increase to 12 (three physicians × four items). If the patient purchased five out-of-pocket expenses, then the number of items in that section would increase to 15 (five expenses × three items). The HAPSQ is designed such that all items are required and must be answered. Therefore, patients are unable to submit their questionnaire until all required items have been answered. This reduces the potential for missing items.

The HAPSQ is a descriptive, health information tool. Therefore, it was not intended to provide one composite score. Instead, items from different sections are combined to provide health information, whereby results can be used to make a judgment about the accessibility, acceptability, efficiency, effectiveness, and safety of care. Table 1 summarizes items in the HAPSQ used to evaluate each of the dimensions. Items measuring waiting times and distance can be used to evaluate accessibility. Items measuring patient satisfaction can be used to evaluate acceptability. Items measuring healthcare consumption, direct costs, and indirect costs can be used to evaluate efficiency. Items relating to patient-suggested waiting times and utilization of healthcare resources can be used to assess appropriateness. Finally, safety can be evaluated by comparing actual clinical care pathways to ideal clinical care pathway algorithms (Eubank *et al*., 2016). In this case, multiple items in the HAPSQ can be combined to map clinical pathways experienced by each patient. Clinical pathways detail steps in the care delivery process of each patient. Therefore, safety can be evaluated by comparing actual clinical pathways to ideal clinical pathway algorithms in order to identify unsafe practices.

**Table 1:** Items from the Healthcare Access and Patient Satisfaction Questionnaire (HAPSQ) mapped to Alberta Quality Matrix for Health’s quality dimensions

Quality dimension	Section	HAPSQ items	Item format
Accessibility	Demographic information	Date of injury*	Date
	Use of physician services	Date of visit*	Date
	Surgery	Date of surgery*	Date
	Use of physician services	Days waiting for consultation*	Numerical
	Use of diagnostic investigations	Days waited before investigation was received*	Numerical
	Demographic information	What is your postal code?	Open-ended
Acceptability	Patient satisfaction rating of care	Overall, how satisfied were you with the quality of care that you received?	VAS scale
	Patient satisfaction rating of care	How satisfied were you with the length of time you had to wait to receive care?	VAS scale
Efficiency	Use of physician services	Have you received care from the following: GP/family physician, sport medicine physician, surgeon, specialist, other?*	Check-box
	Use of physician services	Number of visits*	Numerical
	Use of diagnostic investigations	Have you had any of the following investigations: X-ray, ultrasound, MRI*	Check-box
	Use of diagnostic investigations	Number of investigations*	Numerical
	Surgery	Have you had surgery?*	Check-box
	Use of complimentary allied medical treatments	Have you received treatments from complimentary allied medical providers (eg, physical therapist, chiropractor)?	Check-box
	Use of complementary allied medical treatments	Type of treatment	Open-ended
	Use of complementary allied medical treatments	Number of treatments	Numerical
	Out-of-pocket expenses	Have you purchased out-of-pocket items (eg, braces, pulley, exercise tubing)?	Check-box
	Out-of-pocket expenses	Type of expense	Open-ended
	Out-of-pocket expenses	Number of items	Numerical
	Lost wages	Have you taken time off work?	Check-box
	Lost wages	What is your occupation?	Open-ended
	Lost wages	Full-time or part-time?	Check-box
	Lost wages	Number of days taken off work	Numerical
Appropriateness	Patient expectations around acceptable waiting times	What do you think is an acceptable waiting time (eg, 5 days, 2 weeks, 1 month)?	Numerical

Five demographic questions are not mapped to a quality dimension, are for secondary analysis, and have not been included in Table 1. VAS scale: 100 mm visual analog scale from 0=‘extremely dissatisfied’ and 100=‘extremely satisfied’. Items are combined to map clinical pathways experienced by each patient, which are then used to evaluate the quality dimension: safety.

### Design

Chronic rotator cuff tears were chosen because it ranks among the most prevalent of RCDs that present to the healthcare system (Kemp *et al*., 2011; United States Bone and Joint Initiative, 2014; Tashjian, 2016; Jo *et al*., 2017). Additionally, patients presenting with chronic rotator cuff tears are often treated using conservative, nonoperative management, or surgery (Bokor *et al*., 1993; Kuhn *et al*., 2013; Boorman *et al*., 2014; Kukkonen *et al*., 2015). Therefore, two groups of patients were targeted for this study to provide a representative sample of patients currently presenting to the healthcare system. The inclusion and exclusion criteria for this study are presented in Table 2. Between March and October 2015, a convenience sample of patients presenting with chronic rotator cuff tears was recruited prospectively from the University of Calgary Sport Medicine Centre. Patients were identified from new and follow-up referrals from primary care (eg, emergency room physicians, GPs/family physicians) and sport medicine physicians to three different orthopedic surgeons. Patients eligible for the study were recruited by the primary investigator (B.E.) during scheduled physician appointments. Group 1 included patients that did not require immediate surgical management and were treated conservatively with a nonoperative rehabilitation program (Boorman *et al*., 2014). Group 2 consisted of surgically treated patients who had confirmed surgical dates or had already received surgical management for their shoulder problem. The goal was to first recruit 15 patients for pilot testing the HAPSQ. This sample size was suggested by Zukerberg *et al*. to be optimal for pilot testing (Zukerberg *et al*., 1995). Once pilot testing was completed, recruitment of patients for reliability and validity testing began. The goal was to recruit patients until at least 35 pairs of questionnaires per group was obtained. According to Hertzog, who proposed that for a study to obtain a reliability estimate of at least 0.70, a sample size of at least 35 pairs of questionnaires should be analyzed (Hertzog, 2008). This prospective methodologic study was approved by the Conjoint Health Research Ethics Board at the University of Calgary.

**Table 2:** Inclusion and exclusion criteria

Inclusion criteria	Exclusion criteria
Ages ⩾18 years old English-speaking and literate Chronic, full-thickness rotator cuff tear confirmed by ultrasonography or MRI	Concomitant symptomatic pathology of the affected shoulder (ie, instability, osteoarthritis) Significant cervical spine pathology or radiculopathy Medical gain issues (ie, Workers’ Compensation or litigation) Unable or unwilling to complete study outcomes Unable or unwilling to provide informed consent

### Data analysis

Reliability of the HASPQ was assessed using test–retest reliability and the intraclass correlation coefficient (ICC) for continuous variables of the HAPSQ (13 items). Although there are six forms of ICCs, ICC (2,k) was chosen because it is a measure of agreement between two administrations where the raters are fixed (Shrout and Fleiss, 1979). An ICC of ⩾0.70 was deemed a good measure of reliability (Nunally and Bernstein, 1994). Patients were asked to complete two sets of questionnaires at least one week apart. Any patients with outstanding questionnaires were sent an email reminder at the three-week mark. This was deemed acceptable because the information collected by the HAPSQ was retrospective and thus stable.

Validity of the HAPSQ was assessed through content, criterion, and construct validity testing. Content validity was assessed using two methods. First, patients were interviewed about the relevance and comprehensiveness of the items in the questionnaire. Patients were asked to evaluate the HAPSQ for content, clarity, and readability. Second, content validity was assessed by calculating central tendency, distribution of scores, and floor and ceiling effects for each of the patient satisfaction rating of care items. Large floor and ceiling effects can be an indication that a scale is not valid (Mokkink *et al*., 2010). Patient satisfaction surveys have often been criticized for possessing high ceiling effects (Cappelleri *et al*., 2000). Therefore, 30% was used as the cut-off for acceptable floor and ceiling effects, whereby floor and ceiling effects were indicated if more than 30% of respondents scored the lowest (0) or highest (100) possible score (Kane, 2006).

Concurrent criterion validity was assessed by comparing eight items (eg, items relating to dates, use of physician services, use of diagnostic investigations, and surgery) with patient electronic medical records. The HAPSQ was deemed valid if there was at least 70% agreement with the reference standard (Jonsson and Svingby, 2007).

Construct validity was evaluated through hypothesis testing. Two hypotheses were developed *a priori* and tested. Studies that have analyzed the impact of waiting time on patient satisfaction scores have established that longer waiting times are negatively associated with clinical provider scores of patient satisfaction (Fournier *et al*., 2012; Ansell *et al*., 2017). Therefore, it was hypothesized that patients who experienced longer waiting times to treatment would have an inverse relationship to patient satisfaction scores with respect to time spent waiting for care (Hypothesis 1). Other studies have demonstrated preference in seeking specialist care over primary care for more complex medical conditions because they wanted to obtain the highest quality care (Lewis *et al*., 2000). Therefore, it was also hypothesized that waiting times would have a negligible correlation to patient satisfaction scores with respect to quality of care received because levels of satisfaction were thought to be associated with level of competence in caring for chronic rotator cuff tears (Hypothesis 2). Pearson correlation coefficients were calculated for all hypotheses. An analysis of variance test was used to compare waiting times and patient satisfaction between physician groups.

A *P*-value of <0.05 was considered statistically significant for all analyses. All statistical analyses were performed using SPSS 17.0 software (SPSS Inc., 2007).

## Results

A total of 15 patients were initially recruited for pilot testing the HAPSQ. Once pilot testing was completed, patient recruitment continued until at least 35 Group 1 patients (nonoperative, conservative management) and 35 Group 2 patients (surgical management) completed two sets of questionnaires. A total of 126 patients provided informed consent and were enrolled in the study before 35 pairs of questionnaires in each group were completed. Of these, 13 patients made no attempt to complete the questionnaire and were lost to follow-up, and 17 patients submitted only partially completed questionnaires, in which only the demographic page (Page 1) was completed. Information from these questionnaires was not included in the analysis. Questionnaires from 96 patients were included in validity testing, and questionnaires from 70 patients were included in reliability testing. The patients’ demographic and clinical characteristics are summarized in Table 3. For reliability testing (*n*=70), the average age was 58 years (SD: 9, range: 38–78). The patient population was 64% men (*n*=45), 90% Caucasian (*n*=63), and 26% retired (*n*=18); 39% (*n*=27) of patients reported an annual household income over $100,000. The mean duration of symptoms was three years (SD: 5.0, range: 0.1–25). For validity testing (*n*=96), the average age was 57 years (SD: 10, range: 27–78). The patient population was 62% men (*n*=59), 86% Caucasian (*n*=83), and 23% retired (*n*=22). Of these, 35% (*n*=34) of patients reported an annual household income over $100,000. The mean duration of symptoms was three years (SD: 5.0, range: 0.1–29).

**Table 3:** Patient demographics and clinical characteristics

Variable	Reliability testing (*n*=70)	Validity testing (*n*=96)
Age, mean (SD), years	58 (9)	57 (10)
Age, range (years)	38–78	27–78
Male: *n* (%)	45 (64)	59 (62)
Caucasian: *n* (%)	63 (90)	83 (86)
Retired: *n* (%)	18 (26)	22 (23)
Duration of symptoms: *n* (%)		
<1 year	32 (45)	42 (43)
1–2 years	13 (19)	12 (13)
2–5 years	13 (19)	26 (27)
>5 years	12 (17)	16 (17)
Income: *n* (%)		
<$25,000	3 (4)	6 (6)
$25,000–49,999	5 (7)	6 (6)
$50,000–74,999	11 (16)	14 (15)
$75,000–99,999	9 (13)	12 (13)
>$100,000	27 (39)	34 (35)
Prefer not to say	15 (21)	24 (25)
Treatment: *n* (%)		
Group 1	35 (50)	51 (53)
Group 2	35 (50)	45 (47)

Group 1: Patients that did not require immediate surgical management and were treated conservatively with a nonoperative, rehabilitation program. Group 2: Patients who had confirmed surgical dates or had already received surgery.

Test–retest reliability data for 13 items in the HAPSQ are presented in Table 4. The HAPSQ was completed on an average of 18 days apart (SD: 10, range: 7–39). The ICC (2,k) for various items ranged from 0.60 to 1.0. Only one item failed to reach an ICC>0.70. Out-of-pocket utilization volume possessed an ICC of 0.60. Although an ICC of 0.60 was below the cut-off value of 0.70, there were no other sources of information available to extract patient utilization with respect to out-of-pocket expenses incurred while suffering RCD. Therefore, this item was retained and used in subsequent analyses (Eubank *et al*., 2018). Date of surgery possessed an ICC of 1.0. All Group 2 patients were recruited within 1 year of receiving surgery, and therefore, a perfect score was accepted because it was thought the majority of patients would remember a major life-altering procedure such as surgery.

**Table 4:** Intraclass correlation coefficient (ICC) for continuous variables in the Healthcare Access and Patient Satisfaction Questionnaire

Quality dimension	Section	Item	ICC	95% CI
Accessibility	Demographic information	Date of injury	0.83	0.77–0.86
	Use of physician services	Date of visit	0.99	0.98–0.99
	Surgery	Date of surgery	1.0	1.0–1.0
	Use of physician services	Days waiting for consultation	0.84	0.76–0.89
	Use of diagnostic investigations	Days waited before investigation was received	0.84	0.77–0.89
Acceptability	Patient satisfaction rating of care	Overall, how satisfied were you with the quality of care that you received?	0.85	0.79–0.90
	Patient satisfaction rating of care	How satisfied were you with the length of time you had to wait to receive care?	0.83	0.76–0.87
Efficiency	Use of physician services	Number of visits	0.76	0.65–0.83
	Use of diagnostic investigations	Number of investigations	0.74	0.66–0.80
	Use of complementary allied medical treatments	Number of treatments	0.86	0.77–0.91
	Out-of-pocket expenses	Number of items	0.60	0.47–0.70
	Lost wages	Number of days taken off work	0.82	0.70–0.89
Appropriateness	Patient expectations around acceptable waiting times	What do you think is an acceptable waiting time (eg, 5 days, 2 weeks, 1 month)?	0.93	0.91–0.95

ICC: Intraclass correlation coefficient; CI: confidence interval.

The consensus from patient interviews during pilot testing was that the items in the HAPSQ were mostly thought to be relevant, appropriate, and comprehensive. Twelve patients said that they did not have difficulty completing the questionnaire, and found the questions clear and easy to understand. One patient said that it was hard to rate satisfaction with respect to time spent waiting because in his opinion, waiting for care was unavoidable. One patient was confused about the wording of a question with respect to what it meant by obtaining magnetic resonance imaging (MRI) in the public system or a private facility. This question was reworded to improve clarity and did not cause additional confusion in subsequent pretesting of the HAPSQ. Only one patient noted that it was hard to remember the dates for all of their tests and physician visits. When asked if anyone thought if there were irrelevant questions in the HAPSQ, all 15 patients said ‘no.’ However, two patients did comment that the questionnaire was quite lengthy. The average time it took to complete the HAPSQ was 19 min (range: 14–25 min). No patients suggested adding additional content.

The mean score for the patient satisfaction rating of care item with respect to quality of care was 82.34 (SD: 26.23, range: 0–100). For this item, only 2.1% responded 0 and 28.8% responded 100. The mean score for the patient satisfaction rating of care item with respect to waiting time was 64.89 (SD: 34.47, range: 0–100). For this item, only 4.0% responded 0, and 18.7% responded 100. Applying the 30% cut-off for floor and ceiling effect, neither item demonstrated any floor or ceiling effects.

Criterion validity was evaluated by calculating percent agreement between items relating to dates, use of physician services, use of diagnostic investigations, and surgery with patient electronic medical records. There was evidence of a consultation on the same date for 179/250 (72%) visits upon comparing patient-reported dates and the patients’ medical records. There was also evidence that patients accurately reported rendering 171/210 (81%) physician and diagnostic services. Lastly, there was evidence that 53 (76%) patients accurately reported the number and type of physicians they received care from.

The Pearson correlation coefficient, *r*, was used to evaluate construct validity on two hypotheses developed *a priori*. A significant inverse relationship was found between waiting time and patient satisfaction with respect to number of days waited, thus confirming Hypothesis 1. Specifically, the number of days spent waiting for diagnostic services (*r*=−0.40; *P*<0.001) and physician consultation (*r*=−0.41, *P*<0.001) resulted in lower patient satisfaction scores. There was no relationship between waiting time and patient satisfaction with respect to quality of care received (*r*=0.11, *P*=0.17), thus confirming Hypothesis 2.

Mean patient satisfaction with respect to quality of care was calculated. The mean patient satisfaction score was lowest for emergency room physicians at 67% (SD: 27) and highest for orthopedic surgeons at 89% (SD: 23). Mean patient satisfaction scores for GPs/family physicians and sport medicine physicians were 81 (SD: 26) and 82 (SD: 23), respectively. An analysis of variance demonstrated that patient satisfaction with respect to quality of care provided by a surgeon was significantly different between the other physician groups [F (3, 172)=3.23, *P*=0.02]. Tukey’s highest significant difference *post-hoc* test for significance demonstrated that patient satisfaction for surgeons was significantly higher than emergency room physicians (*P*=0.03).

## Discussion

Assessment of healthcare requires the availability of reliable and valid data on health system performance (Kujala *et al*., 2006). Reliable and valid data have the potential to guide quality improvement activities, redesign services, keep people and organizations accountable for their performance, change policy and practice, and inspire public debate (Leatherman and Sutherland, 2010). In Canada, there are still major gaps between how information on health system performance is measured and monitored by several government agencies and health organizations (Health Council of Canada, 2012). Unfortunately, health services data are inaccurate and difficult to access, thus leaving decision makers with no consistent or comparable set of data to determine the impact of those services (Institute for Clinical Evaluative Sciences, 2012). Therefore, the goal of this study was to evaluate the reliability and validity of the HAPSQ in the context of patients presenting with chronic rotator cuff tears to healthcare settings in Alberta.

Reliability of the HAPSQ was confirmed using test–retest reliability. The ICC for all but one subscale in the HAPSQ was >0.70. Only out-of-pocket utilization volume possessed an ICC of 0.60. Although this may question the validity of this measure, there were no other sources of information available to extract patient utilization with respect to out-of-pocket expenses incurred while suffering a chronic rotator cuff tear.

Content validity was evaluated during pilot testing of the HAPSQ. Results from patient interviews indicated that the HAPSQ was relevant and comprehensive. Content validity was also assessed by calculating central tendency, distribution of scores, and floor and ceiling effects for each of the patient satisfaction rating of care items. Patient satisfaction was used as a measure of the patient’s perception of acceptable care. Studies have expressed concern with using patient satisfaction as a measure of quality, in that many surveys are prone to ceiling effects, which make it difficult to distinguish between the provision of simply adequate services from those providing superior care (Cappelleri *et al*., 2000; Sofaer and Firminger, 2005). Floor or ceiling effects were not found in the HAPSQ. Another criticism to using patient satisfaction is that it only represents one example of a patient perception, and by far, not the only means (Sofaer and Firminger, 2005). Therefore, studies have criticized that in using satisfaction as a measure of quality, one can never be too sure if variations in ratings from one patient to another are the result of differences in expectations or experiences (Sofaer and Firminger, 2005). However, patient satisfaction is a useful determinant in patient outcomes and compliance with treatment (Golin *et al*., 1996; Bartlett, 2002). Additionally, Sofaer and Firminger suggest that asking very specific questions, such as ‘how satisfied they were with the waiting times’ may minimize the subjectivity and the confounding of patient expectations and their ratings (Sofaer and Firminger, 2005). Both patient satisfaction rating of care items in the HAPSQ were specific.

Evidence of concurrent criterion validity was demonstrated when patient-reported data were compared with electronic medical records as the reference standard and percent agreement occurred >70%. Construct validity was tested using two hypotheses developed *a priori*. Both hypotheses were confirmed. The findings that higher waiting times were moderately correlated to lower patient satisfaction scores, but unrelated to the quality of physician care received by the patient both supported construct validity. The findings that patient satisfaction with respect to quality of care were instead associated with the perceived competence level of the caregiver was confirmed, whereby mean patient satisfaction scores were highest for orthopedic surgeons and lowest for emergency room physicians.

Quality of care can be evaluated by collecting adequate, reliable, and valid data using patient self-report measures (Brook *et al*., 2000). The HAPSQ is a tool that gathers quantitative aspects of a patient’s experience for use in evaluating the continuum of care for patients with chronic rotator cuff tears. Analyses of patient-reported outcome measures such as waiting times, patient satisfaction, health resource utilization, and other care processes can then be used to make a judgment about the accessibility, acceptability, efficiency, appropriateness, and safety of care received.

Although there were many strengths to this study, one limitation involved sampling bias. First, the sample population for the study was limited to patients presenting with chronic full-thickness rotator cuff tears. Therefore, the results presented in this study may not be representative of patients presenting with other RCD such as partial-thickness tears, acute tears, or tendinopathy of the rotator cuff. Second, all patients in this study were recruited from sport medicine clinics and seen by an orthopedic surgeon. Therefore, the results may not be generalizable to patients with RCDs that presented to other physician provider groups or complementary allied medical providers.

An argument could also be made about the accuracy of using computer-based patient medical records as a reference standard when assessing criterion validity. The medical record, however, is often viewed as the preferred data source for measuring processes of care and outcome measures (Tisnado *et al*., 2006). Additionally, several studies have demonstrated good to excellent congruency between self-report and electronic medical records (Rozario *et al*., 2004; Tisnado *et al*., 2006).

Despite these limitations, and considering the logistic challenges and increased expenses associated with measuring healthcare quality, the results of this study demonstrate that the HAPSQ represents an inexpensive, reliable, and valid approach toward collecting diagnostic and treatment information across a patient’s continuum of care. The above-mentioned approach to gathering data using the HAPSQ is feasible and likely to succeed. The challenge lies in using the data collected in order to generate additional work to inform practice, research, and policy. As such, there is a demand for reliable, valid, and relevant evidence on which to develop public policy (Swan and Boruch, 2004). Therefore, the development of a tool that can measure healthcare quality is a necessary first step in achieving quality improvements toward healthcare reform.

## Conclusion

This tool represents the first step toward collecting waiting time, resource utilization, patient-reported outcome measures, and cost information at a provincial level for patients presenting to the healthcare system with chronic rotator cuff tears. This study found the HAPSQ to be psychometrically sound. The HAPSQ can thus serve as a cost-effective tool for evaluating health service quality.
